# Spatiotemporal characteristics of elderly population’s traffic accidents in Seoul using space-time cube and space-time kernel density estimation

**DOI:** 10.1371/journal.pone.0196845

**Published:** 2018-05-16

**Authors:** Youngok Kang, Nahye Cho, Serin Son

**Affiliations:** Department of Social Studies, College of Education, Ewha Womans University, Seoul, South Korea; Beihang University, CHINA

## Abstract

The purpose of this study is to analyze how the spatiotemporal characteristics of traffic accidents involving the elderly population in Seoul are changing by time period. We applied kernel density estimation and hotspot analyses to analyze the spatial characteristics of elderly people’s traffic accidents, and the space-time cube, emerging hotspot, and space-time kernel density estimation analyses to analyze the spatiotemporal characteristics. In addition, we analyzed elderly people’s traffic accidents by dividing cases into those in which the drivers were elderly people and those in which elderly people were victims of traffic accidents, and used the traffic accidents data in Seoul for 2013 for analysis. The main findings were as follows: (1) the hotspots for elderly people’s traffic accidents differed according to whether they were drivers or victims. (2) The hourly analysis showed that the hotspots for elderly drivers’ traffic accidents are in specific areas north of the Han River during the period from morning to afternoon, whereas the hotspots for elderly victims are distributed over a wide area from daytime to evening. (3) Monthly analysis showed that the hotspots are weak during winter and summer, whereas they are strong in the hiking and climbing areas in Seoul during spring and fall. Further, elderly victims’ hotspots are more sporadic than elderly drivers’ hotspots. (4) The analysis for the entire period of 2013 indicates that traffic accidents involving elderly people are increasing in specific areas on the north side of the Han River. We expect the results of this study to aid in reducing the number of traffic accidents involving elderly people in the future.

## Introduction

As the economy develops and urbanization rapidly advances in South Korea, an increase in automobile ownership has led to an increase in traffic accidents. In fact, South Korea once recorded the highest traffic accident rate among all OECD nations following increased vehicle ownership resulting from rapid economic growth. However, the number of traffic accidents is now gradually decreasing thanks to various campaigns and preventative actions at the government level [1]. Just as Korea achieved economic growth at an unprecedented rate in the world, its population is also rapidly aging. As of 2015, the proportion of seniors aged 65 or older in Korea accounted for 13.0% of the entire population and, according to a report from the United States Census Bureau in 2016, the proportion of seniors aged 65 or older in Korea is expected to reach 35.9% of the overall population by the year 2050. This number is the second highest in the world after Japan; moreover, Korea’s rate of population aging is reported as being the fastest in the world [2]. Such rapid population aging results in various socioeconomic issues, such as traffic accidents. Although the overall number of traffic accidents is on the decline because of various measures from the government, traffic accidents involving the elderly population (those 65 years of age or older) have increased by 15.8% in 2013 compared to the previous year, and elderly pedestrian traffic accidents are continuously increasing [3]. Therefore, there is need for more focus on the prevention of elderly people-related traffic accidents and for the establishment of countermeasures.

As traffic accidents are human disasters that can be avoided, many studies have analyzed the factors such as age, gender, taking drug, fatigue and weather, etc. that cause traffic accidents [4–9], the factors that have an effect on the level of seriousness of traffic accidents through data mining technique [10–17] and the alternative policy to prevent the traffic accidents [18]. Studies are also being actively conducted on traffic accidents involving seniors as the population rapidly ages. In general, elderly drivers suffer decreased driving abilities and bodily functions as they increase in age, and researches have shown that their probability of dying when a traffic accident occurs is high [19–21]. Studies have also shown that among elderly pedestrians, accidents most often occur close to home and that they are more likely to sustain more serious injuries than other pedestrians [22–24].

Various studies have also analyzed the spatial characteristics of traffic accidents, primarily by analyzing the density on the road network through kernel density estimation or in the form of grid cells in 2D space [25–27]. However, many studies on geovisualization have transformed datasets comprising spatial data such as crime, atmospheric pollution, and diseases into 3D visualizations [28–32]. The existing kernel density estimation method, using 2D point mapping or hotspot analysis techniques, enables simple verification of point data density or clusters, but has difficulty dealing with the time dimension. On the other hand, 3D geovisualization holds the advantage of being able to show spatial patterns, spatial relationships, and changes over time [33–35]. This study visualized and analyzed the spatiotemporal characteristics and changes in traffic accidents involving the elderly population in Seoul using various methods in an effort to help establish preventative countermeasures that can reduce such traffic accidents.

## Materials and methods

### Data

**Fig 1:**
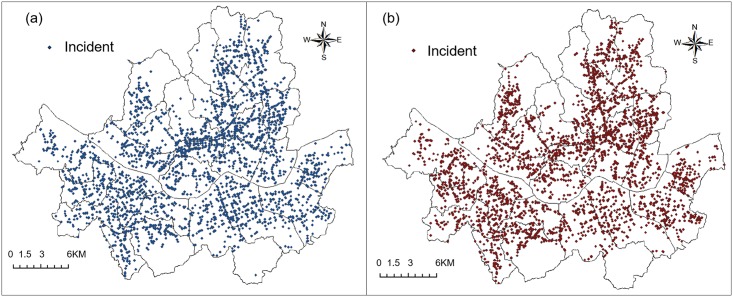
Spots of elderly people’s traffic accidents in Seoul. (a) Elderly drivers; (b) Elderly victims.

In this study, elderly people’s traffic accidents were divided into cases where the senior person was the driver and cases where the senior person was the victim of the traffic accident and analyzed accordingly. The drivers are the people who directly causes the traffic accidents. The victims are the people who are harmed by the traffic accidents. They could be pedestrians, drivers, bicycle riders, and so on. In this study we extracted data on both the drivers and victims who were 65 years of age or older. Traffic accidents involving elderly people that occurred in Seoul during 2013 were used as the data for analysis. These traffic accident data were provided by the National Police Agency of South Korea, and included the location of the traffic accident, driver’s gender and age, victim’s gender and age, time and date of the traffic accident, and weather status. There were 39,439 traffic accidents in Seoul in 2013. The drivers were 65 years of age or older in 3,340 of these accidents, and the victims were 65 years of age or older in 3,590 accidents. Fig 1 shows maps of locations where traffic accidents involving elderly people occurred.

### Analysis

For the analysis process, the text file of traffic accidents provided by the police office was first transformed into GIS data by geocoding the longitude and latitude coordinate information of the location where the traffic accidents occurred. We analyzed the spatial patterns of traffic accidents and then analyzed the spatiotemporal patterns of traffic accidents by adding a time dimension. The spatial patterns were analyzed via kernel density estimation and hotspot analysis. The spatiotemporal patterns were analyzed via hotspot analysis, space-time kernel density estimation (STKDE), and emerging hotspot analysis. Hotspot analysis was employed for hourly analysis, space-time kernel density estimation (STKDE) was applied for monthly analysis, and emerging hotspot analysis was performed to study the trends over time. ArcMap 10.5, ArcPro 1.5, Python’s STKDE source code, and the Voxler program were used to analyze and visualize the data.

#### Kernel density analysis

Kernel density analysis was performed to analyze regions with high density of senior traffic accidents. Kernel density is a method of estimating the spatial density of an entire region based on the distribution of point objects in the target area. It is widely used for visualizing distribution patterns of point data [25, 27, 36]. In kernel density analysis, the visualized data can change according to how the spatial bandwidth is determined and which function is selected. In this study, spatial bandwidth was calculated as follows:

$$SearchRadius\;=\;0.9⨯\text{min}\;\left(SD,\;\sqrt{\frac{1}{\text{ln}\;\left(2\right)}}⨯Dm\right)⨯n^{-0.2}$$

Where:

SD = Standard distance

D_m_ = Average distance

n = Sum of population field values

According to our calculation, the spatial bandwidth was set to 150 meters.

#### Optimized hotspot analysis

Kernel density analysis can show how many traffic accidents occurred within a certain range, but it does not have statistical significance. Therefore, hotspot analysis was performed in order to show the statistical significance regarding regions with high density of senior traffic accidents. Hotspot analysis is useful for analyzing hotspots with high values, coldspots with low values, and for showing which spots have statistically significant patterns. During the analysis of optimized hotspots, all spots are summed in administrative border or grid cell units and analyzed accordingly. In this study we conducted hotspot analysis based on a grid cell of 425 meters. We set up the 425 meters after we created a spatial weight matrix and identified a maximum spatial autocorrelation value, which represents the distances along which those spatial processes are most active. The Getis-Ord Gi* statistical formula was used for hotspot analysis, as follows [37]:

$$G_{i}^{*}\;=\;\frac{\sum_{j\;=\;1}^{n}w_{i,j}x_{j}\;-\bar{X}\sum_{j\;=\;1}^{n}w_{i,j}\;}{S\sqrt[{}]{\frac{\left[n\sum_{j\;=\;1}^{n}w_{i,j}^{2}-{\left(\sum_{j\;=\;1}^{n}w_{i,j}\;\right)}^{2}\right]}{n-1}}}$$

$$\bar{X}=\frac{\sum_{j\;=\;1}^{n}x_{j}}{\text{n}},S=\sqrt[{}]{\frac{\sum_{j\;=\;1}^{n}x_{j}^{2}}{n}-{\left(\bar{X}\right)}^{2}}$$

Where:

*x*_*j*_ = Attribute value for j

*w*_*i*,*j*_ = Weighted value of space between *i* and *j*

*n* = Total number of features

Gi* returns z-score, which represents statistically significant hotspots and coldspots. The values of z-score > 2.58, 1.96 < z-score < 2.58, and 1.65 < z-score < 1.96 represent the hotspots with 99%, 95%, and 90% confidence levels, respectively. The values of the z-score between -1.65 and +1.65 represent no significant cluster. On the other hand, the values of z-score < -2.58, -2.58 < z-score < -1.96, and -1.96 < z-score < -1.65 represent the coldspots with 99%, 95%, and 90% confidence levels, respectively.

#### Space-time cube analysis

Kernel density analysis and hotspot analysis show patterns of traffic accident density, but they do not show the temporal characteristics of these accidents. Thus, for spatiotemporal analysis of traffic accidents, space-time cube analysis was employed. Space-time cube analysis is a 3D geovisualization technique that maps spatiotemporal data in a cube and is useful for finding spatiotemporal patterns. A 3D cube is made up of space-time bins with the x and y dimensions representing space and the t dimension representing time. Every bin has a fixed position in space (x, y) and in time (z). In this study, the value of space (x, y) was 430m x 430m, which was calculated by dividing the longest side of the study area (42750m) by 100.

**Fig 2:**
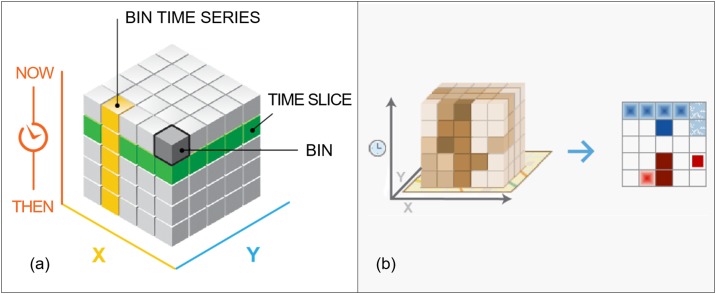
Structure of space-time cube (http://pro.arcgis.com). (a) Space-time bins in 3D; (b) Generated bins in 2D for emerging hot spot analysis.

Two different approaches were used to examine how traffic accident patterns change over time. In the first approach, hourly patterns of traffic accidents were analyzed. As the regions where traffic accidents are concentrated tend to change periodically according to traffic flow of a day, senior traffic accidents locations were analyzed in 24-hour units. To this end, data on the year, month, and day of traffic accidents were excluded such that only the time of occurrence was considered. The temporal and spatial patterns of traffic accidents were then categorized into and analyzed as 24-hour units. A space-time cube was generated by using 24 bins in the z-axis (time), and each bin represents one-hour units; then, hotspot analysis was performed for each period to assess its relevant characteristics (Fig 2).

The second approach is to analyze the trend of traffic accidents that occurred in 2013 by time. In this approach, year, month, and day, as well as time of accident were placed in monthly bins of equal spatial size (430m x 430m). Within each bin, the points were counted and their specified attributes were aggregated. For all bin locations, the trend for counts and summary field values were evaluated and emerging hotspot analysis was performed to analyze the trend by expressing the generated bins in 2D (Fig 2). The hotspots occurrence trend was categorized into 17 different types, including new, consecutive, intensifying, persistent, diminishing, sporadic, oscillating, and historical hotspots and coldspots [38].

#### Space-time kernel density estimation (STKDE) analysis

Space-time cube analysis could be employed to analyze both the split-timed characteristics and the trends of traffic accidents. However, this analysis has a limitation to analyze how the concentrated regions of traffic accidents change by the time. As a result, space-time kernel density estimate(STKDE), developed by Brunsdon et al. (2007), was employed to analyze the changes of the concentrated regions by the time [30, 39]. STKDE values are calculated as follows;

$$\hat{f}(x,y,t)\;=\;\frac{1}{nh_{s}^{2}h_{t}}\sum_{i\;=\;1}^{n}K_{s}(\frac{x-x_{i}}{h_{s}},\frac{y-y_{i}}{h_{s}})K_{t}(\frac{t-t_{i}}{h_{t}})$$

Where:

$\hat{f}(x,y,t)$ = the density estimation at location (*x*, *y*, *t*).

*n* = the number of events

*h*_*s*_ = the spatial bandwidth

*h*_*t*_ is the temporal bandwidth

${(h}_{s}^{2}h_{t})$ is a density value obtained in terms of the number of events per unit space-time by multiplying the density estimate $\hat{f}$ by *n*. The kernel functions *Ks* and *Kt* are defined using the Epanecknikov kernel [40]:

$$K_{s}\left(u,v\right)=\{\begin{array}{ll} \frac{2}{\pi }\left(1-\left(u^{2}+v^{2}\right)\right) & \left(u^{2}+v^{2}\right)<1\\ 0 & otherwise \end{array}$$

$$K_{s}\left(w\right)=\{\begin{array}{cc} \frac{3}{4}\left(1-w^{2}\right) & w^{2}\;<1\\ 0 & otherwise \end{array}$$

Where:

*u* = difference rate between longitude x_i_ and x_i+1_

*v* = difference rate between latitude y_i_ and y_i+1_

*w* = difference rate between time t_i_ and t_i+1_

The STKDE analysis was performed using the STKDE.py Python code developed by Delmelle et al. [41]. The input data are comprised of x, y, and t values in a text file. The spatial bandwidth parameter (h_s) in the Python source code was set to 1 km units, and the temporal bandwidth parameter (h_t) was set to 720-hour units, which is approximately one month. The output results after executing STKDE included z-fields are calculated with the x, y, and t values, and density intensity. The following source codes were used for the STKDE formula, and the results were visualized via the Voxler three-dimensional data visualization and analysis program:

def densityF(x, y, t, x_i_, y_i_, t_i_, n, h_s_, h_t_):

 u = (x-x_i_) / h_s_

 v = (y-y_i_) / h_s_

 w = (t-t_i_) / h_t_

 if pow (u, 2) + pow(v, 2) < 1 and pow(w, 2) < 1:

  constantTerm = pow (10.0, 10) / (n * pow (h_s_, 2) * h_t_)

  Ks = (2 / math.p_i_) * (1 –pow (u, 2)–pow (v, 2))

  Kt = 0.75 * (1 –pow (w, 2))

  spaceTimeKDE = constantTerm * K_s_ * K_t_

 else: spaceTimeKDE = 0

 return spaceTimeKDE

## Results

### Spatial characteristics of senior traffic accidents: Kernel density analysis, hotspot analysis

**Fig 3:**
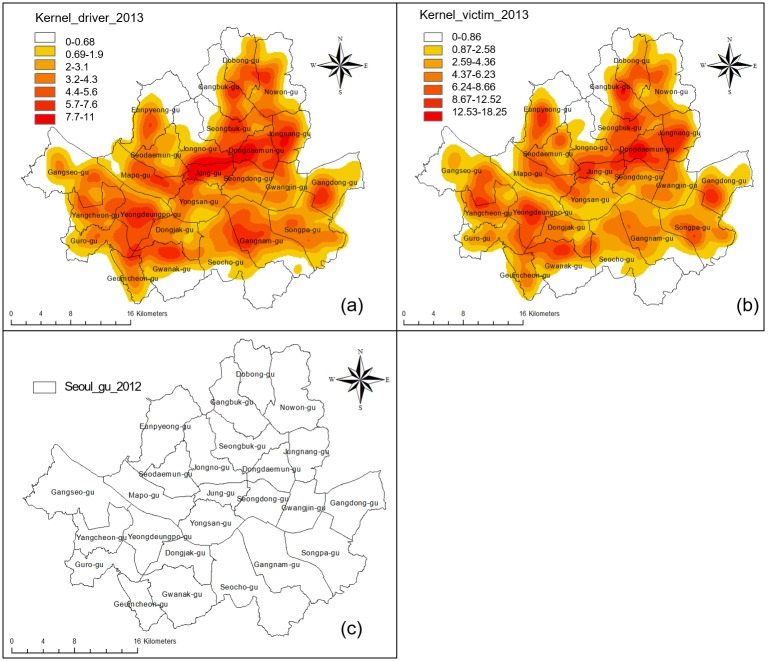
Concentrated regions of elderly people’s traffic accidents using kernel density estimation. (a) Elderly drivers; (b) Elderly victims; (c) Administration boundary.

The areas of concentration of traffic accidents involving the elderly in Seoul during 2013 were analyzed by setting the kernel bandwidth to 150 m. The results showed that traffic accidents were concentrated in Jung-gu, Jongno-gu, Dongdaemun-gu, Jungnang-gu, and Gangbuk-gu. However, identifying major differences between the areas of concentration in terms of cases where the seniors were the drivers and cases where the seniors were the victims of the traffic accidents was difficult (Fig 3).

**Fig 4:**
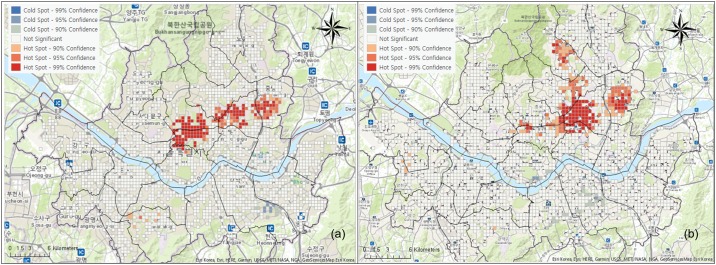
Concentrated regions of elderly people’s traffic accidents using optimized hotspot analysis. (a) Elderly drivers; (b) Elderly victims.

To examine the areas with high concentrations of senior traffic accidents with statistical significance, hotspots and coldspots were analyzed through the z-score distribution of Getis-Ord Gi*. Upon analyzing traffic accident data after converting these data into 425 m cell units, statistically significant hotspot regions where seniors were the drivers in traffic accidents included Jongno-gu, Jung-gu, Dongdaemun-gu, Jungnang-gu, and parts of Seongbuk-gu and Gwanak-gu. Some areas of Gangnam and Songpa-gu included coldspots (Fig 4). On the other hand, hotspots where seniors were the victims of traffic accidents were mainly in Dongdaemun-gu, along with parts of the surrounding Seongbuk-gu, Gangbuk-gu, Jungnang-gu, and Jongno-gu regions. Coldspots were found in some regions of Yongsan-gu, Seocho-gu, and Gangnam. Whereas regions where seniors were the drivers in traffic accidents were concentrated in the northeast urban areas, regions where seniors were the victims were concentrated in the northeast residential areas that were at a slight distance from urban regions. Compared to the results of kernel density analysis on the hotspot regions that were derived through the z-score distribution of Getis-Ord Gi*, regions with statistically higher traffic accidents are more noticeably concentrated.

### Spatiotemporal characteristics of senior traffic accidents

#### Analysis of hotspots by time

**Fig 5:**
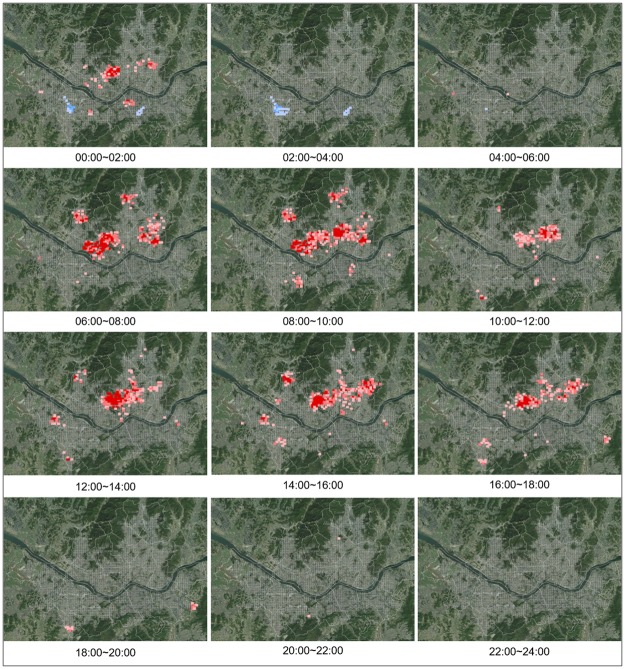
Change of hot spots in 24 hour units in case of drivers.

The analysis results of hotspots and coldspots by time after generating a space-time cube for senior traffic accidents in 24-hour units are shown in Fig 5. The hotspots for traffic accidents with senior drivers were concentrated in Jongno-gu, Jung-gu, and night entertainment areas in some regions of Gangnam during the late hours of the night after midnight, but there was no particular pattern up until 6 a.m. Traffic accident hotspots clearly appear starting from 6 a.m. in Yongsan-gu, Jongno-gu, Seodaemun-gu, and Jungnang-gu, and this pattern persists until 10 a.m. Between 10 a.m. and 12 p.m., traffic accident hotspots typically appear in the urban regions of Gangbuk, and this pattern persists through the afternoon. After 6 p.m., weak hotspots appear in some regions, but they are not very distinct.

**Fig 6:**
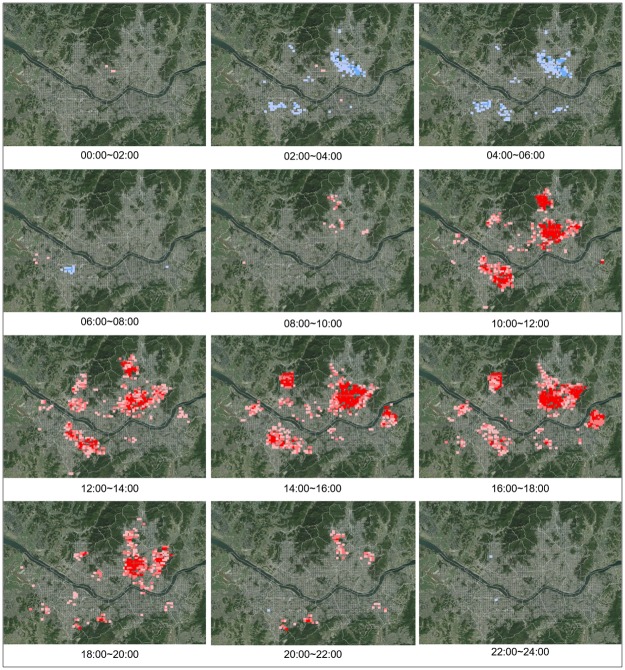
Change of hot spots in 24 hour units in case of victims.

On the other hand, for senior traffic accidents where seniors are the victims, the coldspots appear without any distinct pattern from midnight to 8 a.m., but hotspots begin to appear in some regions of Gangbuk starting from 8 a.m., and similar hotspots appear in Yeongdeungpo-gu, Dongjak-gu, Gangbuk-gu, Dobong-gu, Dongdaemun-gu, and Jungnang-gu from 10 a.m. to 8 p.m. Hotspots also appear in Eunpyeong-gu and Songpa-gu between 2 p.m. and 6 p.m. After 8 p.m., hotspots appear in some residential regions on the outskirts. The analysis results of concentrated traffic accident regions by time provide a greater diversity of data and show that there is clear distinction between patterns where seniors are the drivers and patterns where seniors are the victims (Fig 6).

#### Trend analysis for senior traffic accidents: Emerging hotspot analysis

**Fig 7:**
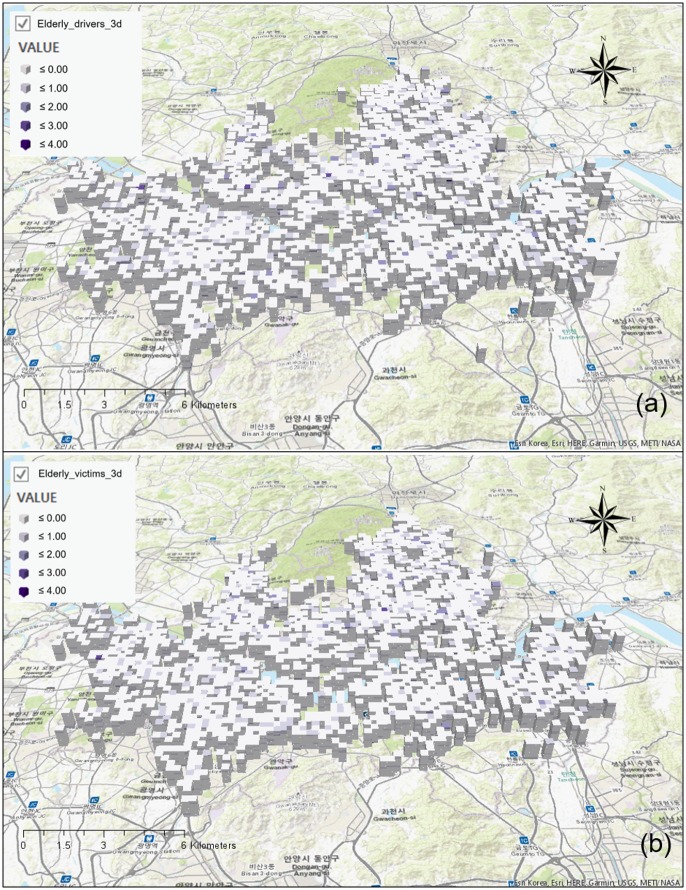
Generated space-time cube for emerging hot spot analysis. (a) Elderly drivers; (b) Elderly victims.

**Fig 8:**
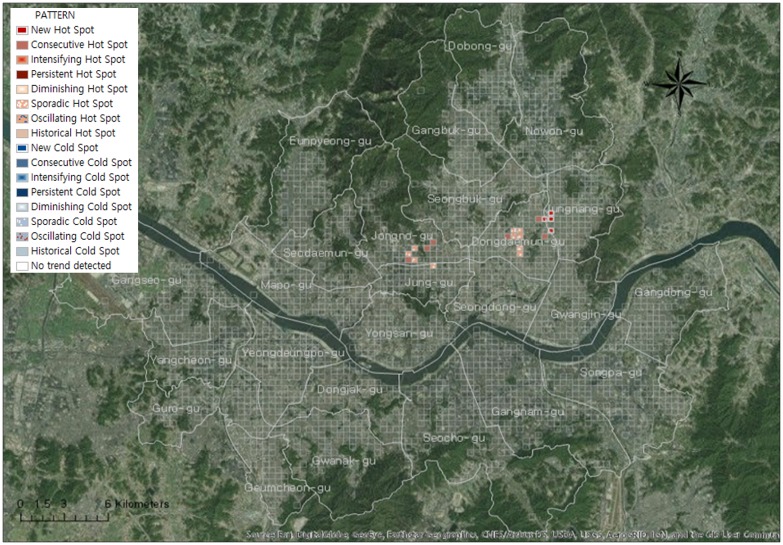
Trends of traffic accidents using emerging hot spot analysis in case of elderly drivers.

**Fig 9:**
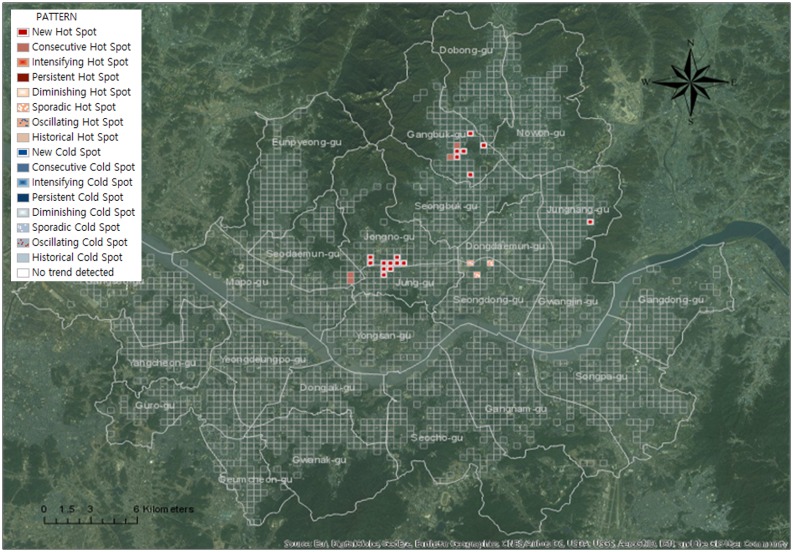
Trends of traffic accidents using emerging hot spot analysis in case of elderly victims.

To analyze the trend of senior traffic accidents in 2013, the spatial unit of the space-time cube was set to 340 × 340 m, and monthly bins were generated for time units. Fig 7 shows the details of the space-time cube that was generated, and Figs 8 and 9 show the details of the analysis conducted of the trend in each bin while expressing the 3D cube two-dimensionally.

Based on the analysis results, there are three types of hotspots for traffic accidents where seniors are the drivers (Fig 8). Three types of hotspots are consecutive hotspots that occurred throughout 2013, new hotspots that appeared as time passed, and sporadic hotspots that repeatedly appeared and disappeared. Consecutive hotspots appeared throughout Cheongnyangni Station in Dongdaemun-gu, near Marronnier Park and Yulgok-ro, and Jonggak Station in Jongno-gu. New hotpots appeared in Jungnangcheon-ro, Jungnang-gyo, and Dongbu Highway in Jungnang-gu. Sporadic hotspots appeared near the University of Seoul, near Deokjeongyo with the intersection between Jeonnong-ro and Wangsan-ro, and near Dapsimni and Dongdaemun Middle School in Dongdaemun-gu.

Three types of hotspots also appeared in areas with a high concentration of traffic accidents where seniors were victims (Fig 9). Consecutive hotspots appeared near Chungjeongno Station and Kyonggi University in Seodaemun-gu, and Samyang-dong and Suyu Market in Gangbuk-gu. New hotspots appeared near Jonggak, throughout Jongno-1,2,3,4 dong, towards Sajik-ro Station in Jongno-gu, throughout city hall and Sogong-dong in Jung-gu, near Mia-dong and Dobong-ro, throughout all of Beon-dong, near Miasageori Station in Gangbuk-gu, and near Myeonilchogyo in Jungnang-gu. Sporadic hotspots appeared at Cheonggyecheon-ro, Seongdong-gu Office, and Majang-ro in Seongdong-gu.

Regions with more concentrated senior traffic accidents in Seoul show a worsening trend in which traffic accidents do not decrease with time, but rather appear continuously or new concentrated regions appear. This is consistent for both traffic accidents that were caused by senior drivers and traffic accidents with senior victims. Analyses of traffic accident trends over time have specified spatial range compared to hotspot analyses that do not consider time.

#### Changes in traffic accident regions: STKDE analysis

**Fig 10:**
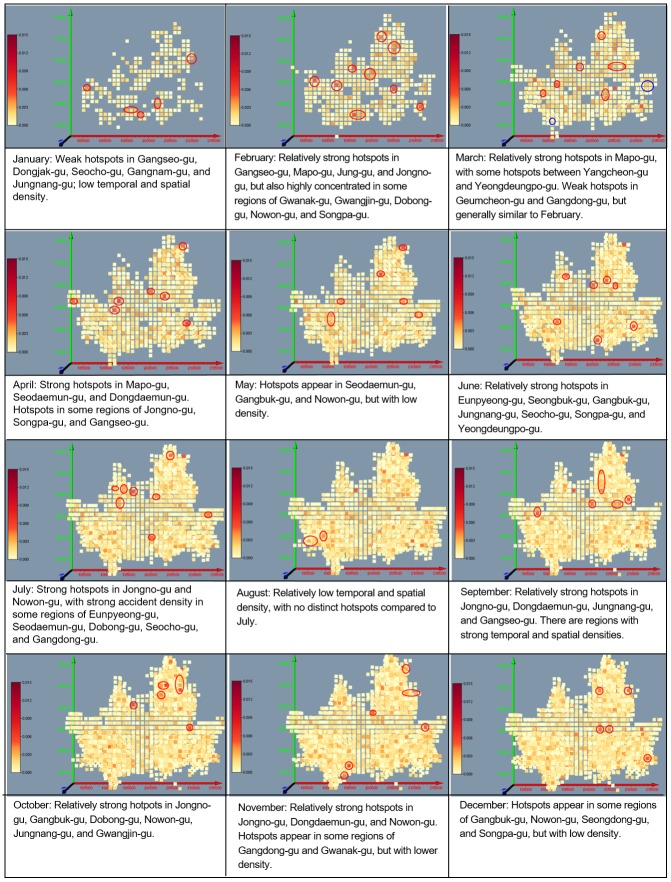
Change of concentrated regions of traffic accidents using STKDE in case of elderly drivers.

**Fig 11:**
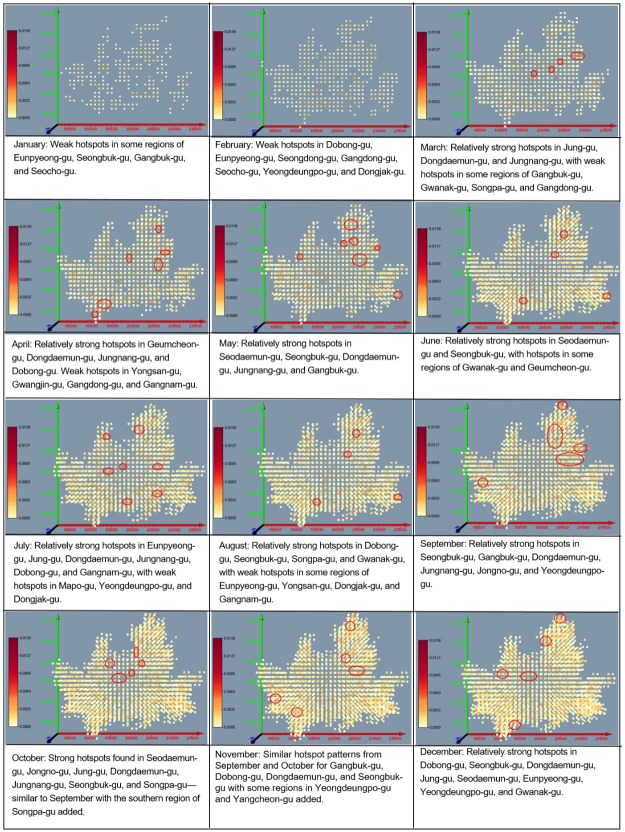
Change of concentrated regions of traffic accidents using STKDE in case of elderly victims.

STKDE analysis was employed to examine the change in traffic accidents over time in a concentrated traffic accident area; it is visualized in Figs 10 and 11. The calculated values of STKDE are divided into five classes by equal interval method. The first and the second classes are defined as strong clusters and the third class is defined as a weak cluster.

In terms of accidents with senior drivers, hotspots appeared mainly in Jongno-gu, Jungnang-gu, Nowon-gu, and Gangbuk-gu, with some hotspots appearing and disappearing in Songpa-gu and Gwanak-gu (Fig 10). Accident density was low in January and February, but relatively strong hotspots appeared in the Gangbuk region in March, April, and May. In July and August, traffic density again decreased, but increased near the mountain regions, such as Bukhan Mountain, Dobong Mountain, and Gwanak Mountain near Seoul, in September to November, when the weather became nicer and people started climbing.

For cases in which seniors were the victims of traffic accidents, hotspots were more sporadic than hotspots where seniors were the drivers (Fig 11). Hotspots were not distinct in January and February, but they appeared in Jongno, Dongdaemun, and Jungnang in March. Hotspots also appeared near the main climbing regions of Seoul, such as Bukhan Mountain in Gangbuk, Dobong Mountain, Inwang Mountain, and Mangweol Mountain, and near Gwanak Mountain in the west, in April and May when the weather became warmer. In June, July, and August, sporadic hotspots appeared in various regions, but hotspots become more concentrated once again in regions with many climbers in Gangbuk in September and October. In November and December, sporadic hotspots appeared rather than being concentrated in specific regions. Upon examining changes in concentrated senior traffic accident regions according to monthly hotspots, it was seen that areas with frequent senior traffic accidents followed seasonal change; there were weak hotspots in the coldest months of January and February, which then became more concentrated in regions that were good for climbing near Seoul during spring and autumn, and again became more sporadic in summer and winter.

## Discussion

This study investigates the spatiotemporal characteristics of traffic accidents involving elderly people by analyzing hotspots over various periods and considering both cases where drivers were elderly people and cases where elderly people were victims. The main findings are as follows: (1) the hotspots for elderly people’s traffic accidents differed between cases in which they were drivers and those in which they were victims. (2) Hourly analysis showed that the hotspots for elderly drivers’ traffic accidents are in specific areas north of the Han River from the morning to the afternoon, whereas the hotspots for elderly victims are distributed over a wide area from daytime to the evening. (3) Monthly analysis showed that the hotspots are weak during winter and summer, whereas they are strong in the hiking and climbing areas in Seoul during spring and fall. Further, elderly victims’ hotspots are more sporadic than elderly drivers’ hotspots. (4) The analysis for the entire period of 2013 indicates that traffic accidents involving elderly people are increasing in specific areas on the north side of the Han River.

Previous studies performed traffic accident analyses considering only cases in which drivers were elderly people or cases in which elderly people were victims of traffic accidents. Moreover, most studies focused on trend analysis in definite periods, even though they tried to analyze the spatiotemporal characteristics of data. In contrast, this study analyzed elderly people’s traffic accidents comprehensively by considering both cases in which drivers were elderly people and cases in which elderly people were the victims of traffic accidents.

In this study we confirm that the hotspots in cases where elderly people are drivers are different from the hotspots in cases where elderly people are victims. Studies have shown that elderly drivers suffer decreased driving abilities and bodily functions, and that elderly pedestrians tend to get severely injured; however, given that elderly people are more susceptible as drivers in some places and more susceptible as victims in others, there need to be different solutions for these discrete issues. We think that the results of our study can help address both situations. For example, the municipal government can take the following steps. In areas in which there are frequent traffic accidents by elderly drivers, the municipal government can dispatch the traffic police or set up the traffic safety facilities. In areas where traffic accidents of elderly pedestrians occur very often, the municipal government can set up safety zones, designate speed limits, or install highlighted warning boards.

However, further research is needed in the following aspects: First, in the analysis of the spatiotemporal patterns of the traffic accident locations, it was thought that allocating the data to a road network would make it easier to analyze the roads where many traffic accidents occurred rather than using grid cells to visualize the data, because traffic accidents occur on the road. Second, the period examined in this study was only the year 2013, which may be considered too short period for comprehensive analysis of temporal and spatial patterns. Hence, it will be necessary to collect and analyze more data over a longer period in the future. Third, after the STKDE analysis, the Voxler program, which visualizes 3D data, was used to visualize the analysis. While this program enables the user to observe data interactively, it comes with limitations with respect to expressing spatial information. Therefore, there were limitations in visualizing the results. The above three limitations must be rectified in future studies.
